# Vitamin D level in COVID-19 patients has positive correlations with autophagy and negative correlations with disease severity

**DOI:** 10.3389/fphar.2024.1388348

**Published:** 2024-05-09

**Authors:** Hongna Dong, Yuqiu Hao, Peng Gao

**Affiliations:** Department of Respiratory Medicine, The Second Hospital of Jilin University, Changchun, Jilin, China

**Keywords:** autophagy, vitamin D, COVID-19, treatment, mechanism

## Abstract

**Background and Objectives:**

There is still incomplete understanding of the pathogenesis of COVID-19. Calcitriol, the main form of vitamin D in serum, regulates immune responses and increases resistance to pathogens, but the mechanism by which it protects against COVID-19 is uncertain. Autophagy has antiviral effects and helps to maintain homeostasis, but its specific role in COVID-19 is also uncertain. Both vitamin D and autophagy have important functions in the lung microenvironment. This study examined the relationship of serum vitamin D and autophagy-related proteins in patients with COVID-19 and evaluated their potential use as biomarkers.

**Methods:**

Blood samples from COVID-19 patients at the Second Hospital of Jilin University were collected. The levels of vitamin D, autophagy-related proteins (Becline 1 [BECN1] and autophagy-related 7 [ATG7]), and inflammatory markers (TNF-α and IL-1β) were measured using enzyme-linked immunosorbent assays.

**Results:**

We examined 25 patients with mild/moderate COVID-19 and 27 patients with severe/critical COVID-19. The group with severe/critical COVID-19 had more abnormalities in many laboratory indicators, including lower levels of autophagy markers (BECN1 and ATG7) and vitamin D, and higher levels of inflammatory markers (TNF-α and IL-1β). Partial correlation analysis showed that vitamin D had strong positive correlations with ATG7 (r = 0.819, *p* < 0.001) and BECN1 (r = 0.900, *p* < 0.001).

**Conclusion:**

Our results demonstrated that the vitamin D level had significant negative correlations with COVID-19 severity and strong positive correlations with autophagy. These findings enhance our understanding of the pathogenesis of COVID-19, and provide a theoretical basis for clinical interventions that target autophagy and vitamin D.

## Introduction

Severe acute respiratory syndrome coronavirus 2 (SARS-CoV-2) is the cause of coronavirus disease 2019 (COVID-19) and the resulting worldwide pandemic. Patients with COVID-19 often experience severe and acute lung injury in addition to other symptoms. There has been progress in the treatment of COVID-19, but mechanical ventilation is required for patients with severe or critical disease status, and these patients have a very high mortality rate (Batah and Fabro, 2021). Therefore, more research, especially the identification of new prognostic biomarkers, is needed to predict patient progression and provide new treatment strategies.

Vitamin D is a cholesterol-derived hormone that plays a role in lung diseases, such as asthma (Chinellato et al., 2011), chronic obstructive pulmonary disease (COPD) (Afzal et al., 2014), pulmonary fibrosis (Chang et al., 2021), lung cancer (Wang et al., 2021), pulmonary cystic fibrosis (Loukou et al., 2020), and hypoxia-induced lung injury (Yao et al., 2017). Vitamin D also has immunomodulatory effects and functions in immune responses (Prietl et al., 2013; Bikle, 2022). In particular, vitamin D regulates the innate and adaptive immune responses during bacterial and viral infections (Bikle, 2008; Telcian et al., 2017). Thus, the role of vitamin D in COVID-19 has attracted widespread attention.

Macroautophagy (autophagy) is an autolysosomal degradation pathway that removes damaged organelles and proteins and is essential for maintaining homeostasis in diverse organisms (Kim and Lee, 2014). Autophagy also plays a role in the immune responses that clear pathogens (Grose, 2010), is essential for lung development and maturation (Yeganeh et al., 2019), and helps maintain the barrier role of lung vascular endothelial cells during lipopolysaccharide (LPS)-induced acute lung injury (ALI) (Zhang et al., 2018). Thus, targeting autophagy may have potential as a therapeutic treatment for ALI. In fact, animal models of LPS -induced ALI showed that genipin, a naturally occurring compound that can cross-link proteins, helped to decrease LPS-induced ALI by promoting autophagy (Zhang et al., 2019). In addition, studies of *in vivo* and *in vitro* models of ALI showed that cinobufagin, another naturally occurring compound, promoted autophagy by activating the p53/mTOR signaling pathway, leading to amelioration of lung permeability and decreased lung inflammation (Wang et al., 2022).

Vitamin D plays a crucial role in lung disease by regulating autophagy. For example, one study that examined an *in vivo* model of respiratory syncytial virus-induced asthma showed that vitamin D mitigated lung tissue damage by inhibiting autophagy (Huang et al., 2022). Another study of mice that examined the fine particle-induced lung injury model found that vitamin D induced autophagy and reduced lung injury by stimulation of the Nrf2 signaling pathway (Tao et al., 2019). Moreover, a study of silica-induced lung injury reported that vitamin D helped to induce autophagy of M2 macrophages and reduce inflammatory damage (Yang et al., 2021). Thus, the results from these studies of different animal models indicate there is a strong link between vitamin D and autophagy.

In the present study, we examined the levels of vitamin D, autophagy related proteins (Beclin 1 [BECN1] and autophagy related-7 [ATG7]), and inflammatory mediators (interleukin-1 beta [IL-1β] and tumor necrosis factor alpha [TNF-α]) in serum samples of patients with COVID-19 and analyzed their correlations with clinical indicators to assess their potential roles in the pathogenesis of COVID-19.

## Materials and methods

### Patient recruitment

The records of 52 adults with COVID-19 who were admitted to the Department of Respiratory and Critical Care Medicine, Second Hospital of Jilin University from December 2022 to March 2023 were retrospectively examined. Twelve healthy volunteers were recruited during the same period. All 52 patients had positive results for SARS-CoV-2 in throat swabs based on real-time reverse transcription (RT) -polymerase chain reaction (PCR), and received treatment with standard protocols. Based on clinical presentation, these patients were classified into four categories: (*i*) mild illness (mild clinical symptoms, no imaging manifestations of pneumonia); (*ii*) moderate illness (fever, respiratory symptoms, imaging findings of pneumonia); (*iii*) severe illness (any one of the following: shortness of breath, respiration rate [RR] of 30 times/min or less, resting pulse oxygen saturation of 93% or less, or partial pressure of oxygen to fraction of inspired oxygen [PaO_2_/FiO_2_] of 300 mmHg or less); or (*iv*) critical illness (any one of the following: respiratory failure or need for mechanical ventilation, shock, or the co-occurrence of failure of other organs and requiring intensive care). This study was approved by the local Ethics Committee (Ethics No. 2023-069) and informed consent was obtained from all study participants.

### Data collection

Basic patient information, medical history, clinical indicators, and other data were obtained from the clinical records. All data were recorded in tabular form for subsequent statistical analysis.

### Blood collection and enzyme-linked immunosorbent assays (ELISAs)

Blood samples were collected upon admission, centrifuged at 3,000 rpm for 20 min, and stored at −80°C prior to testing. The levels of IL-1β, TNF-α, 25-OH-D, BECN1, and ATG7 were measured using human ELISA kits according to the manufacturer’s instructions (Bioswamp, China). First, 50 µL of the standard product with the corresponding concentration gradient were added to the standard wells. Then, a 40 µL sample was added to each sample well, followed by addition of 10 µL of the corresponding biotin-labeled antibody. Second, 50 µL of the enzyme-labeled reagent was added to each well, followed by incubation at 37°C for 30 min. Then, the liquid was discarded, the wells were washed with detergent 5 times, allowed to sit for 30 s, and then patted dry. After that, developer A and developer B were added, mixed by shock, and the color was allowed to develop for 10 min at 37 °C, with blocking from light. Finally, 50 µL of the termination solution was added and absorbance was measured at 450 nm using the Infinite 200 Pro plate reader. The concentration of each sample was calculated according to the standard curve.

### Statistical analysis

SPSS version 21.0 software was used to analyze the data. Means ± standard deviations (SDs) were reported for variables with normal distributions, and medians (interquartile ranges, IQRs) were reported for variables with non-normal distributions. The normality of the data was determined using the Shapiro-Wilk normality test. ANOVA with a least significant difference (LSD) test or Student’s t-test was used for comparisons of data with normal distributions. Some data were subjected to logarithmic transform, and were then analyzed using an LSD *post hoc* test. Data with non-normal distribution were analyzed using the Kruskal–Wallis test followed by the Bonferroni correction or a Mann-Whitney U test for *post hoc* analysis. Spearman’s rank correlation coefficient and partial correlation analysis were used to determine correlations, with adjustment for age and BMI. The threshold for statistical significance was set at *p* < 0.05.

## Results

### Demographic and clinical characteristics of patients

We retrospectively examined the records of 52 patients who were diagnosed with COVID-19 at the Department of Respiratory and Critical Care Medicine of the Second Hospital of Jilin University from December 2022 to March 2023, and recruited 12 healthy volunteers as controls (Table 1). There were 25 patients with mild or moderate disease and 27 patients with severe or critical disease. These two groups had no significant differences in basic characteristics (including age, gender, BMI) or clinical characteristics (including cough, sputum production, fever, and dyspnea). However, the group with severe/critical disease had a longer duration of hospitalization and a worse prognosis (both *p* < 0.05). These two differences were most likely because the severe/critical group had severe or critical lung infection, received long treatment cycles, needed mechanical ventilation (tracheal intubation or tracheotomy), and had low immunity that led to complications and secondary infections that were difficult to treat. Analysis of comorbidities showed that the group with severe/critical disease had a tendency for a greater prevalence of coronary heart disease (*p* = 0.05), a condition that may increase the risk for more severe COVID-19.

**TABLE 1:** Demographic and clinical characteristics of the three groups.

Variable	Healthy (N = 12)	Mild/moderate disease (N = 25)	Severe/critical disease (N = 27)	*P*
Age (years)	64.5(58.3–71.5)	70(59–79.5)	73(67–82)	0.093
Gender (male)	6 (50)	20(80)	21(77.8)	0.127
BMI (kg/m^2^)	22.7(20.4–23.4)	23.5(22.0–26.1)	24.5(20.7–25.7)	0.249
Major symptoms at admission				
Fever		19(76.0)	25(92.6)	0.101
Cough		23(92.0)	23(85.2)	0.447
Expectoration		24(92.3)	19(70.4)	0.050
Chills		1(4.0)	1(3.7)	0.956
Shivering		4(16.0)	7(25.9)	0.386
Fatigue		5(20.0)	7(25.9)	0.616
Thoracodynia		3(12.0)	1(3.7)	0.267
Chest distress		1(4.0)	6(22.2)	0.057
Dyspnea		22(88.0)	20(74.1)	0.207
Sore throat		2(8.0)	3(11.1)	0.707
Headache/dizziness		6(24.0)	3(11.1)	0.224
Nausea/vomiting		2(8.0)	1(3.7)	0.511
Myodynia		7(28.0)	3(11.1)	0.126
Smoking history		7(28.0)	7(25.9)	0.867
Outcomes				
Length of stay, days		13(9-18.5)	21(13-39)	**0.001**
Discharge		25(100)	10(37)	**0.000**
Complications				
Hypertension		15(60.0)	15(55.6)	0.748
Diabetes		6(24.0)	7(25.9)	0.874
Coronary heart disease		2(8.0)	8(29.6)	0.050
Cerebrovascular disease		6(24.0)	7(25.9)	0.672
Liver disease		0(0)	1(3.7)	0.336
Nephropathy		2(8.0)	1(3.7)	0.933
Malignant tumor		0(0)	3(11.1)	0.089

Data are expressed as mean ± SD, or median (IQR). Groups were compared using the Student’s t-test or Mann-Whitney U test. Bold values are statistical difference in *p*-values.

### Laboratory test results

#### Markers of inflammation

We also compared the laboratory results of the two groups (Table 2). The group with severe/critical disease had a greater levels of high-sensitivity C-reactive protein (hsCRP), indicators of coagulation (prothrombin time [PT] and international normalized ratio [INR] of PT), and D-dimer, and lower levels of hemoglobin (HB), red blood cells (RBCs), and platelets (PLTs) (all *p* < 0.05).

**TABLE 2:** Laboratory test results of patients with mild/moderate and severe/critical COVID-19.

Variable	Mild/moderate disease	Severe/critical disease	*P*
White blood cells (10^9^/L)	7.20(5.40–9.00)	7.60(5.60–10.40)	0.912
Neutrophils (10^9^/L)	5.71(4.15–7.97)	5.89(4.44–7.33)	0.840
Lymphocytes (10^9^/L)	0.70(0.60–1.30)	0.60(0.30–0.80)	0.131
Hemoglobin (g/L)	141.00(130.00–154.50)	129.00(93.00–142.00)	**0.014**
Platelets (10^9^/L)	205.00(151.00–278.50)	148.00(85.00–192.00)	**0.017**
Red blood cells (10^12^/L)	4.52(4.29–4.88)	4.11(3.66–4.49)	**0.018**
Fibrinogen (g/L)	4.20(3.80–4.83)	4.39(3.76–4.75)	0.749
D-dimer (µg/mL)	0.86(0.51–1.67)	1.64(1.02–5.08)	**0.005**
Activated partial thromboplastin time (s)	29.50(27.80–32.40)	30.50(27.68–32.85)	0.792
Prothrombin time (s)	11.40(10.75–12.30)	12.35(11.43–13.38)	**0.009**
International normalized ratio	0.96(0.91–1.04)	1.07(0.98–1.13)	**0.005**
Glutamic-pyruvic transaminase (U/L)	33.00(22.50–50.00)	31.50(17.50–77.75)	0.889
Glutamic oxalacetic Transaminase (U/L)	34.00(22.00–43.00)	44.00(26.00–62.00)	0.115
Albumin (g/L)	35.80(33.30–39.90)	32.60(28.35–35.15)	**0.004**
Total bilirubin (µmol/L)	12.00(8.05–13.68)	11.45(9.17–19.37)	0.367
Direct bilirubin (µmol/L)	3.94(2.62–4.45)	5.10(3.52–8.05)	**0.013**
Indirect bilirubin (µmol/L)	8.30(5.10–9.24)	6.61(5.16–9.99)	0.961
Urea nitrogen (mmol/L)	8.18(5.92–9.30)	7.82(7.14–12.82)	0.280
Uric acid (µmol/L)	274.00(190.00–362.00)	259.00(209.00–414.00)	0.985
α1-microglobulin (mg/L)	18.23(14.10–24.43)	19.36(15.00–24.85)	0.756
β2 microglobulin (mg/L)	2.60(1.83–3.91)	3.79(2.93–5.60)	**0.008**
eGFR (mL/min/1.73 m^2^)	84.40(67.00–101.85)	74.30(51.60–94.50)	0.256
Creatine kinase (U/L)	59.00(45.00–83.00)	98.00(37.00–157.00)	0.213
Lactate dehydrogenase (U/L)	270.00(220.00–315.00)	404.00(328.00–598.00)	**0.000**
Hypersensitive C-reactive protein (mg/L)	24.40(7.05–48.28)	57.27(54.54–59.80)	**0.000**
BNP (pg/mL)	47.00(21.93–97.75)	100.50(52.00–561.00)	**0.004**
Troponin (pg/mL)	5.70(2.98–10.20)	15.10(7.60–76.40)	**0.000**
Myoglobin (ng/mL)	28.50(17.10–48.20)	72.80(33.40–165.40)	**0.003**

Data are presented as median (IQR) and groups were compared using the Mann-Whitney U test. Bold values are statistical difference in *p*-values.

#### Markers of liver and kidney function

The group with severe/critical disease had a lower level of albumin, and higher levels of direct bilirubin and β2 microglobulin (all *p* < 0.05).

#### Markers of cardiac function

The group with severe/critical disease also had worse cardiac function, as indicated by higher levels of B-type natriuretic peptide (BNP), troponin, myoglobin, and lactate dehydrogenase (LDH, all *p* < 0.05).

### Vitamin D, markers of autophagy, and cytokines

We then measured the levels of vitamin D two markers of autophagy (ATG7 and BECN1) and two cytokines (TNF-α and IL-1β) (Table 3). Compared with mild/moderate group and the healthy controls, the severe/critical group had significantly lower levels of ATG7, BECN1, and vitamin D, but significantly higher levels of TNF-α and IL-1β.

**TABLE 3:** Serum levels of autophagy markers, vitamin D, and inflammatory factors in the three groups.

Variable	Healthy	Mild/moderate disease	Severe/critical disease	*P*
ATG7 (ng/mL)	21.85(11.96–30.67) ^△※^	12.21(3.72–23.78)^#※^	2.46(2.06–4.79)^#△^	**<0.001**
Beclin-1 (ng/mL)	6.01(4.73–8.86) ^※^	5.55(2.76–7.26) ^※^	2.33(2.05–3.30) ^△#^	**<0.001**
Vitamin D (ng/mL)	27.63(18.74–39.20) ^※^	21.35(9.86–33.02) ^※^	8.16(7.12–12.30) ^△#^	**<0.001**
TNF-α (pg/mL)	101.86(91.97–112.75) ^※^	114.50(86.54–217.89) ^※^	369.53(147.57–763.26) ^△#^	**<0.001**
IL-1β (pg/mL)	687.25(561.92–1,267.51) ^※^	713.29(513.02–992.23) ^※^	1,431.06(625.06–2,691.18) ^△#^	**0.004**

Data are presented as mean ± SD, or median (IQR) and groups were compared using the ANOVA, or Kruskal–Wallis test. ^#^vs. Healthy group, ^△^vs. Mild/moderate group, ^※^vs. Severe/critical group. Bold values are statistical difference in *p*-values.

### Correlation of autophagy markers with vitamin D

**FIGURE 1:**
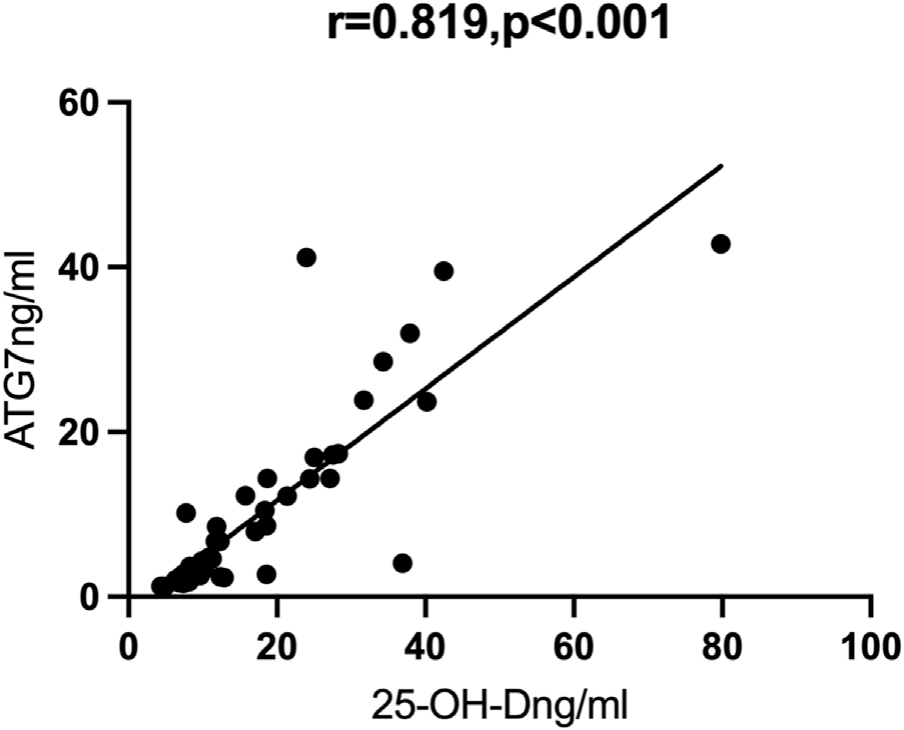
Correlations between ATG7 and 25-OH-D.

**FIGURE 2:**
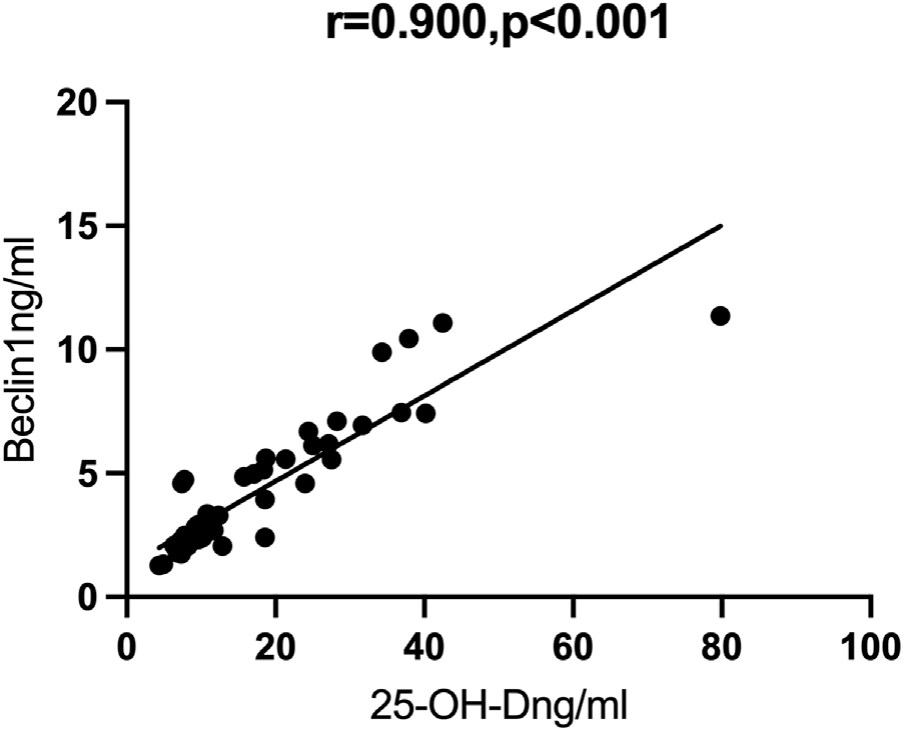
Correlations between Beclin1 and 25-OH-D.

We then performed Spearman correlation analysis to analyze the relationship of vitamin D with these markers. The results showed that vitamin D had significant positive correlations with ATG7 and BECN1, and significant negative correlations with TNF-α and IL-1β (results not shown). We also performed partial correlation analysis with adjustment for age and BMI. The results showed that vitamin D had positive correlations with ATG7 (r = 0.819, *p* < 0.001, Figure 1) and with BECN1 (r = 0.900, *p* < 0.001, Figure 2). In addition, partial correlation analysis also showed that Vitamin D had positive correlations with HB (r = 0.417, *p* < 0.05), RBCs (r = 0.407, *p* < 0.05), albumin (r = 0.379, *p* < 0.05) and negative correlations with IL-1β (r = −0.372, *p* < 0.05), β2 microglobulin (r = −0.337, *p* < 0.05), and clinical classification (r = −0.529, *p* < 0.01) (Table 4).

**TABLE 4:** Partial correlation analysis of autophagy-associated proteins and vitamin D with other indexes, after adjustment for age and BMI.

	ATG7	Beclin1	Vitamin D	Il-1β	Clinical classification	Neutrophils	HB	PLT	RBC	Albumin	β2microglobulin	eGFR	CRP	Myoglobin	Troponin	Length of stay
ATG7	–	0.826 ***	0.819 ***	NS	−0.551 **	NS	0.506 **	0.366 *	0.499 **	0.416 *	NS	NS	−0.443 **	NS	NS	NS
Beclin1	0.826 ***	–	0.900 ***	−0.426 *	−0.575 ***	0.399*	0.436 **	NS	0.442 **	0.374 *	NS	NS	−0.385 *	NS	−0.365 *	−0.372 *
Vitamin D	0.819 ***	0.900***	–	−0.372 *	−0.529 **	NS	0.417 *	NS	0.407 *	0.379 *	−0.337 *	NS	NS	NS	NS	NS

The data were analyzed by partial correlation and adjusted for age and BMI. ***p < 0.001, **p < 0.01, *p < 0.05, NS: not significant (*p* > 0.05).

## Discussion

In the present study of 52 patients with COVID-19, we measured their levels of vitamin D and other disease markers, and assessed the potential function of vitamin D in autophagy and disease severity by comparing two groups of patients: one with severe/critical disease and the other with mild/moderate disease. Our major finding was that the vitamin D level had significant negative correlations with COVID-19 severity, and strong positive correlations with autophagy.

Although our two groups had no significant differences in gender, age, clinical symptoms, or smoking history, the group with severe/critical disease had a longer duration of hospitalization, worse prognosis, and a higher mortality rate. These differences may be because the standard antiviral treatment is less effective in patients with severe illness, because of the difficulty of mechanical ventilation in some of these patients, or because of coinfection by other pathogens, especially drug-resistant bacteria. Coronary heart disease was also more common in patients with severe/critical disease. Laboratory tests showed that the levels of BNP, LDH, troponin, myoglobin, and LDH were significantly elevated in patients with severe/critical disease. An elevated level of serum LDH is associated with an increased risk of cardiovascular disease (Zhu et al., 2022). Consistent with previous studies, we found that COVID-19 patients with severe/critical disease had a significantly increased risk of heart damage (Huang et al., 2021). The co-occurrence of coronary heart disease with COVID-19 is an indicator of poor patient prognosis (Liang et al., 2021). An elevated level of serum LDH on admission is a predictor of mortality in patients who present with severe/critical COVID-19 and with cardiovascular complications (Dong et al., 2020; Masumoto et al., 2022). Because COVID-19 can lead to heart damage, it is especially important to monitor indicators of heart function so that timely treatment can be administered.

Our results also suggested that although our two groups had no significant differences in leukocyte count or neutrophil count, the group with severe/critical disease had decreased levels of HB, RBCs, and PLTs. Patients with COVID-19 often have abnormal blood clotting indicators (Levi et al., 2020; Wool and Miller, 2021). In agreement, our results showed that the group with severe/critical disease had an abnormal INR, a greater level of D-dimer, and a longer PT time. These abnormalities in coagulation and fibrinolysis are associated with poor prognosis in patients with COVID-19 (Liu et al., 2020; Lazzaroni et al., 2021). More specifically, D-dimer is an indicator of poor outcome from COVID-19 (Poudel et al., 2021) and a prolonged PT is a predictor of intensive care unit (ICU) admission and poor prognosis in patients with COVID-19 (Baranovskii et al., 2021). Early identification of abnormalities in coagulation may therefore help to assess the risk of thrombosis, so that effective treatment measures can be rapidly administered.

A low level of albumin (indicating impaired synthesis) and a high level of direct bilirubin (indicating increased excretion) are indicators of abnormal liver function, and our severe/critical group had abnormal levels of these two markers. However, our two groups did not have significant differences in glutamic pyruvic transaminase or glutamic oxalacetic transaminase. Previous studies of COVID-19 showed that albumin was an important prognostic factor, and that a higher baseline level was associated with a decreased risk of adverse events, such as venous thromboembolism, acute respiratory distress syndrome, ICU admission, and mortality during hospitalization (Kheir et al., 2021). This suggests that clinicians should be alert to liver injury in patients with COVID-19, and should pay attention to changes of albumin and other markers of liver function to improve the long-term outcome of these patients.

Blood β2-microglobulin is an important indicator of renal function. This protein functions in the development of emphysema (Gao et al., 2017) and is a marker of pulmonary fibrosis in patients with COPD (Wu et al., 2020). We found that the level of β2-microglobulin was significantly higher in the group with severe/critical COVID-19. Gong et al. reported similar results in COVID-19 patients who were infected with the Omicron variant of SARS-CoV-2 (Gong et al., 2023). Other studies of patients with COVID-19 also showed that β2 microglobulin was an indicator of more severe disease, poor prognosis, and COVID-19-associated kidney injury (Fukao et al., 2021), (Conca et al., 2021). Therefore, the increase of β2-microglobulin may predict the deterioration of renal function and the aggravation of pulmonary fibrosis, and help predict the development of COVID-19.

Our patients with severe/critical disease had increased levels of multiple inflammatory markers (TNF-α, IL-1β, and hsCRP), indicating more severe inflammation and even the possible presence of a cytokine storm. Specific biological therapies, such as tocilizumab and infliximab, have great potential for treatment of COVID-19 patients with severe inflammation (Rommasi et al., 2022). Elevated levels of CRP and D-dimer in patients with COVID-19 are also associated with an increased risk of thrombosis (Dujardin et al., 2020), poor prognosis, acute kidney injury, venous thromboembolism, death, and progression to critical illness (Smilowitz et al., 2021).

Our most important findings were related to vitamin D. Vitamin D is a cholesterol-derived hormone and an important immunomodulator (Luo et al., 2020; Dhawan et al., 2022). Previous studies showed that vitamin D plays an indispensable role in the immune responses against pulmonary viral infections. For example, a study of mice with lungs that were infected with the H1N1 virus and SARS-CoV-2 demonstrated that vitamin D reduced inflammation and played a protective role (Arora et al., 2022). Consistent with previous studies, we found lower levels of vitamin D in patients with severe/critical COVID-19 (Nielsen et al., 2022). Another study of elderly COVID-19 patients reported that vitamin D deficiency was associated with poor prognosis, severity of lung disease, and duration of disease (Sulli et al., 2021). There is also evidence that a low level of vitamin D can predict the degree of fibrosis in patients with idiopathic pulmonary fibrosis (Tzilas et al., 2019).

Vitamin D is also known to promote autophagy, a process that is essential for lung development and morphological maintenance (Yeganeh et al., 2019). In addition, autophagy can inhibit the production of inflammasomes and IL-1β, and prevent hypoxemia and increased lung permeability caused by ALI, thereby protecting lung structure and function (Nosaka et al., 2020). Our study showed that the levels of vitamin D and two autophagy markers (ATG7 and BECN1) were lower in patients with severe/critical COVID-19 than in those with mild/moderate COVID-19. Other research showed that vitamin D induced autophagy and prevented particulate and silica-induced ALI (Tao et al., 2019; Yang et al., 2021). Our partial correlation analysis showed that vitamin D had strong positive correlations with two autophagy markers (ATG7 and BECN1), suggesting a close link between vitamin D and autophagy in COVID-19. However, further *in vivo* and *in vitro* studies of this relationship and the underlying mechanism are required.

One limitation of this study is that we only examined the levels of circulating autophagy marker proteins. It is likely that these circulating markers had lower sensitivity and specificity than samples collected directly from the lungs for the characterization of lysosome function and the level of autophagy in lung tissues. However, due to the difficulty in obtaining lung tissue from COVID-19 patients, we were unable to analyze lung tissues. We performed a preliminary exploration of the effects of vitamin D and autophagy on COVID-19, and provided new insights into the possible mechanism and treatment of COVID-19. In the future, it is necessary to elucidate this molecular mechanism in animal models and cell experiments to provide more definitive evidence of potential therapeutic targets. In addition, our study was conducted in a single center with a small patient sample size, and therefore could have been affected by sampling bias and limited external validity. In the future, it is necessary to expand the sample size and conduct a larger multicenter cohort study to verify the reliability and stability of the results presented here.

## Conclusion

This study examined the relationship between the serum level of vitamin D and autophagy in patients with COVID-19 by comparing patients with mild/moderate disease and those with severe/critical disease. Our results suggest that a low level of vitamin D may lead to inhibition of autophagy, and thereby contribute to the pathogenesis of COVID-19. More in-depth studies that confirm this relationship and further examine the mechanism of vitamin D and autophagy may be helpful for the development of novel adjuvant therapeutics for COVID-19.

## Data Availability

The original contributions presented in the study are included in the article/Supplementary Material, further inquiries can be directed to the corresponding author.

## Abbreviations

ALI, acute lung injury; ATG7, autophagy related-7; BNP, B-type natriuretic peptide; COPD, chronic obstructive pulmonary disease; COVID-19, coronavirus disease 2019; ELISAs, enzyme-linked immunosorbent assays; HB, hemoglobin; hsCRP, high-sensitivity C-reactive protein; IL-1β, interleukin-1 beta; INR, international normalized ratio; IQRs, interquartile ranges; LDH, lactate dehydrogenase; LPS, lipopolysaccharide; PLTs, platelets; PT, prothrombin time; RBCs, red blood cells; RT-PCR, reverse transcription-polymerase chain reaction; SARS-CoV-2, severe acute respiratory syndrome coronavirus 2; SDs, standard deviations; TNF-α, tumor necrosis factor alpha.
